# Measuring healthy ageing: current and future tools

**DOI:** 10.1007/s10522-023-10041-2

**Published:** 2023-07-13

**Authors:** Nádia Silva, Ana Teresa Rajado, Filipa Esteves, David Brito, Joana Apolónio, Vânia Palma Roberto, Alexandra Binnie, Inês Araújo, Clévio Nóbrega, José Bragança, Pedro Castelo-Branco, ALFAScore Consortium, Raquel P. Andrade, Sofia Calado, Maria Leonor Faleiro, Carlos Matos, Nuno Marques, Ana Marreiros, Hipólito Nzwalo, Sandra Pais, Isabel Palmeirim, Sónia Simão, Natércia Joaquim, Rui Miranda, António Pêgas, Ana Sardo

**Affiliations:** 1 Algarve Biomedical Center Research Institute (ABC-RI), Campus Gambelas, Bld.2, 8005-139 Faro, Portugal; 2 ABC Collaborative Laboratory, Association for Integrated Aging and Rejuvenation Solutions (ABC CoLAB), 8100-735 Loulé, Portugal; 3 Faculty of Medicine and Biomedical Sciences (FMCB), University of Algarve, Gambelas Campus, Bld. 2, 8005-139 Faro, Portugal; 4 Department of Critical Care, William Osler Health System, Etobicoke, ON Canada; 5 Champalimaud Research Program, Champalimaud Centre for the Unknown, Lisbon, Portugal

**Keywords:** Healthy ageing, Ageing scores, Ageing biomarkers, Biological age

## Abstract

Human ageing is a complex, multifactorial process characterised by physiological damage, increased risk of age-related diseases and inevitable functional deterioration. As the population of the world grows older, placing significant strain on social and healthcare resources, there is a growing need to identify reliable and easy-to-employ markers of healthy ageing for early detection of ageing trajectories and disease risk. Such markers would allow for the targeted implementation of strategies or treatments that can lessen suffering, disability, and dependence in old age. In this review, we summarise the healthy ageing scores reported in the literature, with a focus on the past 5 years, and compare and contrast the variables employed. The use of approaches to determine biological age, molecular biomarkers, ageing trajectories, and multi-omics ageing scores are reviewed. We conclude that the ideal healthy ageing score is multisystemic and able to encompass all of the potential alterations associated with ageing. It should also be longitudinal and able to accurately predict ageing complications at an early stage in order to maximize the chances of successful early intervention.

## Introduction

### Ageing and healthy ageing

The term “healthy ageing” has been widely used to describe high-functioning older adults based on their physical and mental attributes. Initially, healthy ageing was felt to preclude chronic disease (Rowe and Kahn 1997)**.** However, recently, there has been a shift from a disease-centred model of healthy ageing towards a function-centred paradigm (Cesari et al. 2018; Cosco et al. 2014). The World Health Organization (WHO) characterises healthy ageing as the “process of developing and maintaining a functional ability that enables well-being in old age” (World Report on Ageing and Health 2015). Functional ability depends on the intrinsic capacity (IC), which is the sum of the individual’s physical and mental competencies, as well as the individual’s environment and risk factors (World Report on Ageing and Health 2015).

The remarkable increase in human longevity observed during the last century has led to a substantial increase in the number of elderly individuals alive today (Vaupel 2010). This has been accompanied by an increase in the prevalence of numerous chronic, non-communicable diseases that arise in old age, such as cardiovascular disease, cancer, osteoarthritis, and diabetes mellitus type II as well as neurodegenerative diseases such as Alzheimer’s Disease and Parkinson’s Disease (Franceschi et al. 2018; Li et al. 2021). In fact, the main risk factor for the development of these diseases is age itself (Hayflick 2021). Understanding the fundamental biology of ageing is necessary but difficult to achieve, since the progression, rate and phenotype of ageing differs among organism, organ, cell types, and molecules within a cell (Rattan 2018). At the cellular level, the molecular hallmarks of ageing include compromised cell and tissue function. These cellular effects lead to systemic age-related pathologies that are accompanied by loss of function and, ultimately, death (López–Otín et al. 2013; Schmauck-Medina et al. 2022; Singh et al. 2019a, b).

Most ageing-related diseases have long latent periods that precede their disease manifestations. In the early stages of the disease, buffering at the molecular level, delays their influence on phenotype and functional status. However, when perturbations reach a certain severity, they eventually cause clinically-measurable changes in anatomic and physiological parameters, limiting physical and cognitive function (Ferrucci et al. 2018).

This buffering capacity is the homeodynamic space of a biological system, determining an individual’s health, and the ability to survive and maintain a healthy state. The extent of homeodynamic space achieved by an individual depends both on genetic factors and on pre-natal and early-life epigenetic factors, including nutrition, infections, mental stimulation and physical activity (Rattan 2013, 2020).

Currently, the best strategies to increase healthspan are physical exercise, healthy nutrition, and life in a socially supportive environment (World Health Organization 2012). Health-oriented and preventive strategies, such as hormesis: heat/cold exposure, dietary restriction, exercise, and cognitive stimulation, have proven to be approaches that potentiate the homeodynamic space and delay ageing (Rattan 2012). However, these strategies are not always sufficient to ensure healthy ageing and are difficult for many individuals to sustain. Thus, there is significant interest in developing new therapies to promote healthy ageing, and simultaneously implement tools to monitor and evaluate their efficacy.

## Ageing scores

Chronological age only partially reflects an individual's functional and health characteristics. Ageing scores are used in epidemiological and sociodemographic settings to characterise the health status of a population (Rodriguez–Laso et al. 2018) Scores typically include chronological age, sex, race, lifestyle, body composition, and the presence of chronic diseases as well as other quantifiable phenotypical or clinical inputs (Newman et al. 2008). The data used to derive ageing scores are taken from epidemiological studies that may be longitudinal, following the same individuals over time, or cross-sectional, evaluating individuals at a single time point. Scores are typically created by weighting the factors/variables according to their impact on the intended outcome, whether this is physical or cognitive performance, disease risk, or mortality. Scores are calculated by factor analysis or by obtaining sub-scores for specific domains based on the distribution of the sample, such as z-scores, quartiles and categories. The combination of the sub-scores is then achieved by arithmetic sum or average. In combination with socio-economic indicators, scores can ascertain the long-term impact of socio-economic and educational factors, lifestyle behaviours, and occupational risks on the ageing quality of a population (Dieteren et al. 2020; Liu et al. 2019; O’Connell et al. 2019). In the clinical context, ageing scores can help determine an individual’s disease risk, providing a nuanced view of their ageing status and potentially guiding early interventions for at-risk individuals. Numerous ageing scores have been proposed, which can be loosely divided into three subtypes: (a) phenotypic, (b) functional and (c) biological (Ferrucci et al. 2018).

The choice of derivation cohort affects the applicability of a given score to individuals. Thus, it is crucial that ageing scores are validated in multiple populations to ensure generalized application (World Report on Ageing and Health 2015).

Despite the change in paradigm of ageing, still in present days epidemiologically viable metrics of ageing biology are mostly based on factors which reflect an individual’s organismal deterioration. The frailty index (FI) is one of the main methods of clinical evaluation to assess the quality of ageing (Searle et al. 2008). It represents the proportion of accumulated deficits of an individual using 40 variables (symptoms, signs, functional impairments and laboratory abnormalities), reflecting the severity of illness and proximity to death (Mitnitski et al. 2001).

### Physiological and phenotypic healthy ageing scores

The first ageing scores were based on physiological and phenotypic parameters. The *Physiological Index of Comorbidity* (PIC) (Newman et al. 2008) score was designed to identify subjects who were at medium to low risk of disease for enrolment into clinical trials (Charlson et al. 1986). It was based on a combination of clinical measures to identify underlying disease risk. These included: carotid ultrasound, pulmonary function testing, brain magnetic resonance (MRI) scan, serum cystatin-C, and fasting glucose levels. The PIC has been validated as a predictor of mobility limitation, difficulties with activities of daily life (ADL), and mortality. The *Healthy Ageing Index* (HAI) (Sanders et al. 2014) is a simplification of the PIC that replaces the brain MRI with a cognitive performance test - the Mini-Mental Status Exam (MMSE) - and the carotid ultrasound with systolic blood pressure. The HAI is used extensively in epidemiological studies to characterise and compare the ageing of different populations worldwide (Nie et al. 2021; O’Connell et al. 2019; Wu et al. 2018; Zhang et al. 2021).

Modified versions of the HAI have also been created by adding other variables, particularly functional dimensions, to quantify the impact of lifestyle and life experience on ageing quality (Table 1). The *Successful Ageing - Health domains* score was derived from an exploratory factor analysis that identified the domains of healthy ageing and their predictive factors (Mount et al. 2019). Authors found that Insulin Growth Factor 1 (IGF-1) levels and arterial pulse pressure (the difference between systolic and diastolic blood pressure) were predictors of neuro-sensory functional decline. Similarly, the *Biological Health Score* [(Karimi et al. 2019), Table 1] is designed to measure the “wear and tear” of ageing and includes biological markers from 4 physiological domains (endocrine, inflammatory, cardiovascular and metabolic) and two organs (liver and kidney). The *Biological Health Score* was used to examine the impact of socio-economic position (SEP) on biological ageing, showing that education-related differences could be detected even in young adults (20–40 years old), making a case for the early application of a HAS.

**Table 1:** Scores of healthy ageing and intrinsic capacity

Name of score	Objective	Variables	Outcome
Healthy ageing index (Dieteren et al. 2020)	Identify ageing trajectories and evaluate the role of baseline sociodemographic characteristics and lifestyle factors. Longitudinal study	Systolic blood pressure, non-fasting plasma glucose levels, global cognitive functioning, plasma creatinine levels and lung functioning	Classification in 'early' and “gradual’ ageing population. Lifestyle factors (e.g. nutrition and physical activity) appear to play an important role in optimal ageing
Chinese healthy ageing index (CHAI) (Nie et al. 2021)	Creation of a composite measure of healthy ageing in the Chinese population. Investigate changes in the index over time. Longitudinal study	Blood pressure, peak expiratory flow, cognitive status score, fasting glucose, kidney function and C-reactive protein	Index range (0–12), from healthiest to unhealthiest
Successful ageing—Health domains (Mount et al. 2019)	Use exploratory factor analysis to identify domains of healthy ageing. Longitudinal study	Physical function, cognitive status, social interactions, psychological status, blood biomarkers, disease history, and socioeconomic status allowed the identification of 4 domains of ageing: neuro-sensory function, muscle function, cardio-metabolic function, and adiposity	Prediction of objective but not subjective measures of successful ageing. IGF-1 and pulse pressure levels are related to neuro-sensory function decline
Biological Health score (Karimi et al. 2019)	Create a score capturing the wear-and-tear of four physiological systems and determine the impact of SEP on biological ageing. Cross-sectional study	Endocrine: DHEAS, testosterone (men); Inflammatory: CRP, fibrinogen, and IGF-1; Metabolic: A1C, HDL, total cholesterol, and triglycerides; Cardiovascular: systolic and diastolic blood pressure, pulses. Liver: ALT, AST and GGT; Kidney: creatinine*	Contribution of the inflammatory and metabolic systems to the overall score. Physiological differences can already be observed in the early-adult group (20–40 years)
Universal Healthy ageing scale (Sanchez-Niubo et al. 2021)	Creation of a universally applicable scale to evaluate healthy ageing and ageing trajectories classification. Cross-sectional study	16 worldwide cohorts (343.915 individuals). 41 items encompassing activities of daily living and cognitive and physical functioning. Scores were rescaled according to the cohort	Association with various sociodemographic, life and health factors and healthy life expectancy. Classification in 3 ageing trajectories
Intrinsic capacity (Yu et al. 2021)	Examine the structure and predictive capacity of the ICC. Longitudinal study	ICC domains: Locomotor, vitality, sensory, cognitive, psychological	Prediction of incident IADL limitations at the 7-year follow-up
Multidimensional model of healthy Ageing (Rivadeneira et al. 2021)	Applying the ICC, identify indicators that discriminate healthy ageing from less healthy ageing. Cross-sectional study	a) ICC domains: physiological and metabolic health, geriatric syndromes, risk factors, physical capacity, cognitive capacity, and psychological well-being. b) social and political environment. c) the interaction of the older adult with the environment	Gender and economic situation seem to play an important role in healthy ageing
Intrinsic capacity (Cheong et al. 2022)	Create ICC index. Explore the performance of combining domain-specific measures. Cross-sectional study	ICC domains using different variables of: locomotor vitality, sensory, cognitive, psychological	Validity of 3-domain ICC using Time Up-and GO + LogMAR (visual) + ENIGMA (nutritional). Showed excellent correlations with known health determinants
Intrinsic capacity (Gutiérrez-Robledo et al. 2019)	Describe the levels of intrinsic capacity and factors related to its decline. Cross-sectional study	ICC domains: cognition, depression, hearing, vision, anorexia, weight loss, and mobility	Decreased levels of intrinsic capacity were associated with less schooling, self-rated health, chronic diseases, visits to a physician, and ADL

*Dehydroepiandrosterone sulfate (DHEAS), C-Reative protein (CRP), Insulin growth factor - 1 (IGF-1), Hemoglobin A1C (A1C), high-density lipoprotein cholesterol (HDL), Alanine transaminases (Alt), Aspartate aminotransferase (Ast), Gamma-glutamyl transpeptidase (Ggt)

Following an appeal from the WHO to improve harmonisation between ageing scores, the *Universal Healthy Ageing Scale*, was derived from a harmonized dataset created from 16 worldwide longitudinal cohorts, called the “Ageing Trajectories of Health: Longitudinal Opportunities and Synergies” (ATHLOS) dataset [(Sanchez–Niubo et al. 2021), Table 1]. It is hoped that the application of the *Universal Health Ageing Scale* will help to harmonise future ageing studies globally.

Recently, the *Intrinsic Capacity Construct* (ICC) was proposed, which takes a slightly different view of ageing based on the concept that, although an individual's functional capacity may have fallen below its peak, they may still be able to maintain key functions if they live in a supportive environment (Cesari et al. 2018). The ICC comprises 5 domains: cognition, psychological, locomotion, sensory and vitality. The ICC has been validated in populations around the world [(Cheong et al. 2022; Gutiérrez-Robledo et al. 2019; Rivadeneira et al. 2021; Yu et al. 2021), Table 1], showing that it can predict Instrumental Activities of Daily Living limitations at 7-year follow-up (Yu et al. 2021) and has an excellent correlation with other known health determinants (Cheong et al. 2022). However, the components of the ICC differ amongst studies (Table 1), making cross-study comparisons difficult. Standardisation and further validation are necessary to make the ICC a relevant and useful tool in the clinical and community setting (George et al. 2021; Rivero-Segura et al. 2020). Despite the change in paradigm of ageing, still in present days epidemiologically viable metrics of ageing biology are mostly based on factors which reflect an individual’s organismal deterioration. The frailty index (FI) is one of the main methods of clinical evaluation to assess the quality of ageing (Searle et al. 2008). It represents the proportion of accumulated deficits of an individual using 40 variables (symptoms, signs, functional impairments and laboratory abnormalities), reflecting the severity of illness and proximity to death (Mitnitski et al. 2001).

### Biological ageing scores

Although physiologic and phenotypic ageing scores are useful for assessing the health and functional status of elderly individuals at a specific point in time, the window for disease prevention or behaviour correction may already have closed. Consequently, there is significant interest in identifying early predictors of healthy ageing that can be measured and compared at any age (Hartmann et al. 2021; Justice et al. 2018; Lohman et al. 2021).

“Biological age” (BA), is conceptualized as a surrogate measure of a healthy lifespan at any age (Kwon and Belsky 2021). The heritable contribution to lifespan is estimated to be only 25–30% (Brooks-Wilson 2013; van den Berg et al. 2017), as shown by studies of monozygotic twins (Zenin et al. 2019), as well as populations living in the “blue zones” of healthy ageing, which include Okinawa in Japan, Sardinia in Italy, and Nicoya in Costa Rica (Buettner and Skemp 2016). In fact, genome-wide association studies (GWAS) have identified only a few loci that are consistently linked with longevity and healthspan, such as apolipoprotein E (ApoE), Forkhead Box O3 (FOXO3), LDL Receptor Related Protein 1B (LRP1B) and Cyclin-Dependent Kinase Inhibitor 2A/B (CDKN2A/B) (Deelen et al. 2019; Melzer et al. 2020). Thus, researchers have turned to physiological variables and multi-omics markers to help explain the observed variation in healthspan.

Biological ageing scores are derived from ageing datasets that typically include demographic data, outcome data - functional and physiological, and multi-omics data – epigenetics, transcriptomics, proteomics, metabolomics and microbiome data. Machine learning (ML) approaches allow hypothesis-free data mining of these large datasets and can model many different dimensions of the ageing process (Farrell et al. 2022; Kwon and Belsky 2021). ML network analysis enables the connection between different types of information, and the relationships between different dimensions may represent effects which cannot be described just by statistical correlations (Dato et al. 2021). These approaches have led to the development of biological ageing scores based on a variety of data types as well as the concepts of ageing phenotype, ageing trajectory, and ageotype, discussed below.

The difference between BA and chronological age (CA) may be positive, indicating accelerated biological ageing, or negative, indicating decelerated (or healthy) biological ageing. The ideal marker of biological age should provide reliable prognostic information about future ageing-associated outcomes including comorbidities, functional status or mortality. It should be able to predict disease onset in pre-symptomatic individuals and identify causal lifestyle behaviours, aiding in the development of disease prevention strategies (Belsky et al. 2018).

### Physiological ageing scores

Several biological ageing scores have been derived using physiological variables. *PhenoAge* is an ML derived biological ageing score that captures morbidity and mortality risk across diverse populations, independent of chronological age (Levine et al. 2018; Liu et al. 2018). It comprises 10 physiological variables and is strongly associated with future disease count (Table 2), enabling researchers to evaluate the benefits of early interventions. *Biological Age* is the product of an ML approach in which investigators used a deep neural network (DNN) to identify blood biomarkers of healthy ageing [(Gialluisi et al. 2022), Table 2]. The strongest markers of mortality and hospitalisation risk were Cystatin-C, N-terminal-pro hormone B-type natriuretic peptide (NT-proBNP), and gender. The *Physiological Ageing* score (PA) [(Sun et al. 2021), Table 2] was derived from two independent cohorts of individuals in long-lived communities (SardiNIA and InCHIANTI). The ratio of PA to chronological age (PAR) was found to be a significant predictor of survival as well as a proxy for whole-body ageing. The ATHLOS harmonised dataset modelled individual healthy ageing trajectories over 10 years [(Nguyen et al. 2021), Table 2], defining 3 ageing trajectories: a 'high stable’ group, a 'low stable’ group, and a ‘rapid decline’ group. Abstinence of physical activity and specific multimorbidity patterns were associated unfavourable ageing trajectories (Moreno-Agostino et al. 2020; Nguyen et al. 2021).

**Table 2:** Scores measuring ageing rates and biological age

Name	Objective	Variables	Outcome
PhenoAge (Levine et al. 2018)	Determine the applicability for differentiating risk for various health outcomes within diverse subpopulations that include healthy and unhealthy groups and distinct age groups. Cross-sectional study	Chronological age, albumin, creatinine, glucose, CRP, % lymphocyte, mean Red blood cell volume and distribution, weight, alkaline phosphatase, and White blood cell count	A biological age measure; highly predictive of mortality and independent of chronological age. Strong association with disease count. Used as a base for the DNAmPhenoAge clock
Biological age (Gialluisi et al. 2022)	Biological age algorithm using DNN. Longitudinal study	36 clinical biomarkers and gender	Δage (chronological age—biological age) significantly predicted mortality and hospitalisation risk. Major contributors to BA were cystatin-C, NT-proBNP and gender. A decelerated BA was associated with higher physical and mental well-being, healthy lifestyle and higher socioeconomic status, while accelerated ageing was associated with smoking and obesity
Physiological ageing rate-PAR (Sun et al. 2021)	Predict physiological ageing rate from quantitative traits. Identify genetic loci by GWAS. Longitudinal study	ML analysis of 148 variables in the InCHIANTI and sardiNIA ageing cohorts. GWA	Predictor of physiological age. Major contributors are pulse mean velocity, CCA intima-media thickness, peak systolic velocity, diastolic CCA diameter, waist circumference and BMI. If PAR > 1, the individual’s physiological age is greater than their chronological age. GWAS 2 loci associated with PAR: CFI/GAR1, LINC00202
Universal Healthy ageing trajectories (Moreno-Agostino et al. 2020; Nguyen et al. 2021)	Describes healthy ageing trajectory patterns and association with multimorbidity. Determine the impact of groups of diseases over ageing trajectories. Longitudinal study	41 items related to health and functioning, such as ADL cognitive and physical functioning, using data from 7 harmonised cohorts	Definition of 3 ageing patterns: "high stable", "low stable", and "rapid decline" groups. The cardiorespiratory/arthritis/cataracts population group was associated with the "rapid decline" and the "low stable" groups

### Epigenetic biological age

Epigenetic biological ageing scores, also known as epigenetic clocks, are collections of DNA methylation sites whose aggregate methylation status measures age (Hannum et al. 2013; Horvath 2013). The most commonly applied clocks are the blood-based algorithm by *Hannum* (Hannum et al. 2013) and the multi-tissue algorithm by *Horvath* (Horvath 2013). Both produce a DNA methylation (DNAm) age that correlates very closely with CA (r = 0.94). Researchers have hypothesized that deviations from CA observed in epigenetic clocks may reflect BA and health status. Second-generation or “composite” epigenetic clocks include a larger number of DNA methylation sites and also incorporate DNAm surrogates of ageing biomarkers previously described (Bergsma and Rogaeva 2020; Simpson and Chandra 2021). The *DNAmPhenoAge* epigenetic clock (Levine et al. 2018) was created by regressing a physiological measure of mortality risk – *PhenoAge* – on DNA methylation markers [(Levine et al. 2018; Liu et al. 2018), Table 3]. Increased *DNAmPhenoAge* was associated with increased activation of pro‐inflammatory and interferon pathways as well as decreased activation of transcriptional/translational machinery, DNA damage response, and mitochondrial signatures, suggesting that these pathways are important in ageing (Levine et al. 2018). *DNAmGrimAge* is based on surrogate DNAm markers of seven plasma proteins that increase with age as well as DNAm markers of smoking [(Lu et al. 2019a), Table 3]. *DNAmGrimAge* was shown to predict longevity and was also sensitive to age-related pathologies, including cognitive decline (Hillary et al. 2021), depression (Protsenko et al. 2021), hypertension (Robinson et al. 2020), and long-term cardiovascular health (Joyce et al. 2021). In The Irish Longitudinal Study on Ageing (TILDA, N = 590), *DNAmGrimAge* outperformed *Horvath*, *Hannum*, and *DNAmPhenoAg* epigenetic clocks in predicting all‐cause mortality and age‐related clinical phenotypes.

**Table 3:** Composite Next-Generation blood epigenetic clocks used in healthspan ageing research

Epigenetic clock of ageing	Method CpG sites	Additional variables	Outcome
DNAm PhenoAge (Levine et al. 2018)	Illumina 450 K 513 CPGs	DNAm surrogate of PhenoAge: chronological age, albumin, creatinine, glucose, C-reactive protein, lymphocyte %, mean red blood cell volume, red blood cell distribution weight, alkaline phosphatase, White blood cell count	DNAm PhenoAge is moderately heritable and is associated with activation of pro-inflammatory, interferon, DNAm damage repair, transcriptional/ translational signalling, and various markers of immuno-senescence: a decline of naïve T cells and shortened leukocyte telomere length
DNAmGrimAge DNAmGrimAgeAA (Lu et al. 2019a)	Illumina 450 K&Epic 1030 CPGs	DNAm based surrogates: ADM, B2M, Cystatin-C,GDF-15, Leptin, PAI-1, TIMP-1, DNAm based estimator of smoking pack-years* DNAmGrimAgeAA: DNAmGrimAge and chronological age	Lifespan predictor. Results are given in years. High predictive ability for time‐to‐death. DNAm-based surrogate biomarker for smoking pack-years is a better predictor of mortality than the self-reported biomarker. Associated with age-related changes in blood cell composition and leukocyte telomere length. Correlated with lifestyle factors and a host of age-related conditions
DNAmTL DNAmTLadjAge (Lu et al. 2019b)	Illumina 450 K&Epic 140 CPGs	Developed by regressing measured Leucocyte TL on blood methylation DNAmTLadjAge**:** DNAmTL and Chronological age	Leukocyte DNAmTL has a strong association with several ageing-related diseases, physical fitness/functioning, dietary variables, educational attainment, and income. DNAmTLadjAge is heritable and significantly associated time-to-death, all-cause mortality, time-to-CV disease, later age at menopause and positive association with physical activity
DunedinPace, Pace of Ageing Calculated from the Epigenome (Belsky et al. 2022)	Illumina Epic 173 CPGs Longitudinal study	DNAm surrogates of 19 indicators of organ-system integrity: BMI, Waist-hip ratio, A1C, Leptin, BP, VO_2_Max, FEV1/FVC, FEV1, Total cholesterol, Triglycerides, HDL, Lipoprotein(a), ApoB100/A1 ratio, eGFR, BUN, hs-CRP, White blood cell count, mean periodontal attachment loss, tooth decay**	Added incremental prediction of morbidity, disability, and mortality beyond DNAmGrimAge. Can be used to complement previously generated epigenetic clocks
mDNAage (Vetter et al., 2022a)	Illumina Epic MS-SNuPE 7 CPGs	Chronological age and leukocyte cell distribution	Adaptation and development of a cost-effective epigenetic clock base on 7 CpGs. Applicable to 2 sequencing techniques

*Adrenomedullin (ADM), beta-2-microglobulin (B2-M), growth differentiation factor 15 (GDF-15), Plasminogen activator inhibitor 1 (PAI-1), and tissue inhibitor metalloproteinases 1 (TIMP-1). **Body mass index (BMI), Hemoglobin A1C (A1C), Blood pressure (BP), Maximal oxygen consumption (VO2Max), Forced expiratory volume (FEV1), Forced vital capacity (FVC),   Apolipoprotein B (ApoB100), Apolipoprotein A1 (ApoA1), estimated glomerular filtration rate (eGFR), blood urea nitrogen (BUN), high-sensitivity c-reactive protein  (hs-CRP)

The ideal epigenetic clock should also detect the beneficial effects of an improved lifestyle. In a 2-year follow-up, *DNAmGrimAge* detected alterations in the quality of dietary consumption (Fiorito et al. 2021). Similarly, the *Horvath* epigenetic clock showed evidence of deceleration with improved dietary, lifestyle behaviours, and medication (Fahy et al. 2019; Fitzgerald et al. 2021; Gensous et al. 2020). Although these are small studies with only short-term follow-up, these results suggest that epigenetic clocks could be useful in assessing the efficacy of preventative strategies or treatments to decrease ageing rate or modify ageing trajectories.

An alternative epigenetic strategy for measuring BA is to analyse DNAm associated with telomere shortening, one of the hallmarks of cellular ageing. Leucocyte telomere length (TL) has been widely studied as an ageing biomarker (Vaiserman and Krasnienkov 2021). However, discrepancies in measurement methodologies and issues with replicability have undermined its utility (Lulkiewicz et al. 2020). The *DNAmTL* is an epigenetic clock that indirectly measures telomere length (Lu et al. 2019b). This method is easier to use and more robust than standard TL measurements (Table 3) and is more sensitive to age-related conditions such as disease and physical fitness, making it a potentially useful biomarker in ageing interventional studies.

DNA methylation is dynamic, so longitudinal studies are necessary to understand how DNAm changes during the life of an individual. A promising next-generation DNA-methylation biomarker was recently developed using data from the Dunedin Study 1972–1973 birth cohort (Belsky et al. 2020), which includes 4 longitudinal timepoints. The Dunedin-Pace of Aging Calculated from the Epigenome Score (*DunedinPACE*) [(Belsky et al. 2022), Table 3]. is based on DNAm surrogates for 19 physiological markers of organ-system integrity (Dieteren et al. 2020; Sanders et al. 2014; Wu et al. 2017) combined with DNAm markers of periodontal attachment loss and tooth decay. The last two variables were incorporated to reflect lifestyle and income, socioeconomic factors that have been linked to ageing quality. The use of longitudinal cohort data to build the *DunedinPACE* score helped eliminate many potential confounding factors, including survival bias (Vrijheid 2014), and has been validated in 5 epidemiological studies showing improved prediction of morbidity, disability, and mortality when compared to *DNAmGrimAge* (Belsky et al. 2022). The cost-effectiveness of the epigenetic clock are also a requirement for future clinical application, the mDNAage clock [(Vetter et al. 2022a), Table 3] was developed based on 7CpGs only and is sensitive to cardiovascular health scores (Lemke et al., 2022).

Although studies suggest that DNAm clocks can predict future disability and mortality, the benefit of epigenetic clocks over more traditional phenotypical and physiological ageing scores is still uncertain. A validation of 5 DNAm clocks using data from the Berlin Aging Study II failed to show an association between DNAm results and health deterioration or loss of function after 7 years (Vetter et al., 2022b). In a separate study, markers of epigenetic age acceleration were unable to predict a change in frailty at 1.5 years of follow-up (Seligman et al. 2022). Further validation is necessary to determine whether DNAm clocks can accurately predict functional outcomes, during long-term follow-up (Föhr et al. 2022; Maddock et al. 2020). Intriguingly, healthy individuals display methylation changes that are associated with both accelerated and decelerated epigenetic ageing. Thus, an individual’s epigenetic age is a function of the relative contribution of each site to their overall DNA methylation profile (Shahal et al. 2022).

### Transcriptomic biological age

The transcriptome is the collection of all messenger RNA (mRNA) transcripts expressed from the genes of an organism. It is a dynamic entity, which varies between cell types and changes rapidly in response to developmental and environmental cues. Using whole-blood gene expression data, Peters et al. identified 1497 genes whose expression was associated with chronological age in leukocytes. The authors used gene expression profiles to calculate a *Transcriptomic Age* and showed that differences between CA and *Transcriptomic Age* were associated with important biological features of ageing including blood pressure, serum cholesterol, fasting glucose, and body mass index (Peters et al. 2015). More recently, a Self-Organizing Maps ML (SOM-ML) analysis of whole blood transcriptome data [(Schmidt et al. 2020), Table 4] revealed two major blood transcriptome types. Type 1 was characterized by increased inflammation and increased heme metabolism and was more commonly found in men, older individuals, and obese individuals. Type 2 was characterised by transcriptional activation and immune activation and was more commonly found in women, younger individuals, and normal weight individuals.

**Table 4:** Biological Age determination using omics and multi-omics tools

Name Reference	Methodology	Objectives	Variables	Outcome
Blood Transcriptome (Schmidt et al. 2020)	Whole blood transcriptome, microarray analysis	Characterising the diversity of transcriptional states and their impact on cellular functions and association with ageing phenotypes	Lifestyle, obesity, disease history, medication status and age	Identified 2 main blood transcriptomes, whose signatures were shaped by immune response and inflammatory processes
BitAge (Meyer and Schumacher 2021)	RNA-seq, human dermal fibroblast	Develop a transcriptomic ageing clock base on *c. elegans* but applicable to human fibroblast transcriptome data	Age and a progeria syndrome group	Longitudinal in *C. elegans*. Validated in human fibroblasts showing contribution of the innate immune response, neuronal signalling, and single transcription factors for biological age
ProAge, PROAge Accel (Tanaka et al. 2020, 2018)	Plasma proteome 76 proteins Longitudinal	Develop a method for in-depth diagnostic procedures and early interventions in ageing. Used only healthy adults	Age, disease and mortality	Identification of a 76-protein proteomic age signature *PROAge*, predictive accumulation of chronic diseases and all-cause mortality. Development of *PROAgeAccel* for ageing rate quantification
Proteome Ageing clock (Lehallier et al. 2020)	Plasma proteome 491 proteins	Datamining of protein patterns. Reactome pathway analysis	Age and lifestyle	Proteins associated with signal transduction, or the immune system are capable of predicting human age. Aerobic-exercised trained individuals have a younger predicted age than sedentary subjects
Metabolomic age (Robinson et al. 2020)	Urine and serum metabolome	Determine metabolomic age. Relate the metabolome with determinants of accelerated ageing	Lifestyle and psychological risk factors for premature mortality	Correlated with chronological age. metabolic Age Acceleration (mAA) was related to overweight/obesity, diabetes, heavy alcohol use and depression
The plasma metabolome (Johnson et al. 2019)	Plasma metabolome by LC–MS	Identify plasma metabolomic signatures associated with biological ageing in healthy adults	Klemera and Doubal biological age. 360 plasma metabolites	Plasma metabolites are predictive of faster vs. slower ageing trajectory. Metabolites most associated with the rate of biological ageing include amino acid, fatty acid, acylcarnitine, sphingolipid, and nucleotide metabolites
Microbiome clock (Galkin et al. 2020)	Stool 13 Illumina datasets ENABrowser	Gut Microbiome Ageing Clock Based on Taxonomic Profiling and Deep Learning	Age and disease	Prediction of host age from gut microflora profiles. The clock is sensitive to disease presence. Could be used as a starting point for anti-ageing intervention design
Biological age (Earls et al. 2019)	Multi-omics Longitudinal study	Biological age estimation, applying the Klemera-Doubal algorithm using deep phenotyping variables	Genetic, clinical lvalues, metabolome, and proteome	Measures of metabolic health, inflammation, and toxin bioaccumulation were strong predictors of increased BA over time
Ageotype (Ahadi et al. 2020)	Multi-omics Longitudinal study	Use deep phenotyping to find a measure correlated with age	Transcriptomics, proteomics, metabolomics, cytokines, microbiome, and clinical laboratory values	Individuals were grouped in ‘ageotypes’, based on the types of molecular pathways that changed over time in a given individual
Archetype (Zimmer et al. 2021)	Multi-omics Longitudinal study	Generate individual archetypes. Find enriched traits for each archetype by deep phenotyping	Lifestyle,Fitbit records, genomics, microbiome, metabolomics, and proteomics	The model can be used for early detection of transitions from health to disease state, identify aberrant health conditions and ageing

Tools to determine age in vitro are also required to facilitate the study of cellular mechanisms of ageing and in vitro testing of anti-ageing therapies. The *Binarized Transcriptomic Aging Clock* (BiT age) [(Meyer and Schumacher 2021), Table 4] is a transcriptional clock that was developed in *C. elegans* and validated in human fibroblasts, where it showed a high degree of accuracy in predicting BA. The genes included in *BiT age* support roles for transcription factors, the innate immune response and neuronal signalling as key pathways in cellular ageing (Gill et al. 2022; Meyer and Schumacher 2021).

### Proteomic biological age

Proteins are appealing as biomarkers of ageing because their role as direct biological effectors makes it likely that they will reflect the physiological changes of ageing (Tanaka et al. 2018). Recently, a plasma proteomic signature of age, *PROAge*, was designed to identify individuals who were ageing faster than their CA [(Tanaka et al. 2020), Table 4]. *PROAge* includes 76 ageing-associated proteins and predicts the development of both ageing-associated diseases and mortality. A second, ultra-predictive ageing clock was generated that included 491 plasma proteins [(Lehallier et al. 2020), Table 4]. This clock predicted a younger BA for individuals who did regular exercise relative to those who were sedentary. Proteins associated with the immune system were particularly useful in predicting CA and BA.

### Metabolomic biological age

The metabolome, defined as the collection of small molecules, and their interactions, within a biological system, is altered during ageing and may reflect underlying physiological function (Johnson et al. 2019). Plasma metabolome analysis by ultra-high performance liquid chromatography–mass spectrometry (UHPLC-MS) was used to identify metabolites that predict faster biological ageing [(Johnson et al. 2019), Table 4]. The metabolites most strongly associated with ageing included amino acid, fatty acid, acylcarnitine, sphingolipid and nucleotide metabolites. In a separate study, metabolomic predictors of age were identified in urine and blood samples from a longitudinal UK cohort and validated in a longitudinal Finnish cohort [(Robinson et al. 2020), Table 4]. Accelerated metabolomic age, defined as metabolomic age greater than CA, was associated with obesity, diabetes, alcohol use, and depression (Robinson et al. 2020).

A new approach to metabolomics is the analysis of volatile organic compounds (VOCs), low-weight carbon-based molecules that can be detected in sweat, exhaled breath, blood, urine, and faeces. Urinary and faecal VOCs can distinguish different age groups and can also discriminate the offspring of centenarians from age-matched controls (Conte et al. 2021, 2020).“Breathomics” is the quantification of VOCs in breath samples. In one study, it was shown to detect age-related differences amongst females (Sukul et al. 2022). The use of VOCs as biomarkers of ageing requires further validation, however, the development of a non-invasive tool to monitor ageing and/or age-related conditions would be invaluable.

### Microbiome measurements in ageing

The gut microbiome is responsible for diverse biological and metabolic functions, including vitamin synthesis, digestion of dietary fibre, and regulation of the host immune response (Adak and Khan 2019; Knight et al. 2017). To assess an individual’s microbiome, next generation sequencing is applied to faecal samples to measure the frequency of ribosomal RNA markers that are specific to certain microbes or groups of microbes. Machine learning techniques are then applied to the sequencing data to identify features associated with ageing. The top predictor of longevity in older age groups is alpha-diversity, a measure of within-sample diversity (Biagi et al. 2016; Kong et al. 2016). However, clustering of individuals based on alpha-diversity is difficult because the microbiome becomes increasingly divergent and unique with age. This individual uniqueness is associated with the enrichment of health-associated bacteria and may be a favourable adaptation to ageing (Biagi et al. 2016; Kong et al. 2016; Wilmanski et al. 2021).

Studies have shown associations between the makeup of the gut microbiome and diet, physical fitness, and frailty, all of which affect health span (Jackson et al. 2016). Differences in the prevalence of specific microbial species have also been associated with key markers of health, including inflammation, diastolic blood pressure, and weight, suggesting that the microbiome plays a role in healthy ageing (Claesson et al. 2012). A recent study comparing the gut microbiome of healthy and unhealthy older adults reported an abundance of *Akkermansia* and *Erysipelotrichaceae* taxa in the healthy cohort. The authors hypothesised that these fermentative, complex carbohydrate-digesting bacteria promote healthy intestinal barrier function and thereby contribute to healthy ageing (Singh et al. 2019a, b). In contrast, the *Enterobacteriaceae* family have been associated with mortality risk in the general population over an extended follow-up (Salosensaari et al. 2021).

The association between microbe prevalence and ageing recently led to the development of a microbiome-based ageing clock [(Galkin et al. 2020), Table 4]. The taxa that were most predictive of CA were *Bifidobacterium spp.*, *Akkermansia muciniphila*, and *Bacteroides spp,* which were associated with good ageing quality, and *Escherichia coli* and *Campylobacter jejuni*, which were associated with poor ageing quality. Notably, most microbes only impacted age prediction when their relative abundance reached a minimum threshold, suggesting that low threshold microbes play a limited role.

In addition to reflecting the health of the host, microbiome composition determines microbial metabolic outputs that are subsequently absorbed by the host. These can induce physiological responses and also impact the host metabolome (Lozupone et al. 2012). Indeed, proteomic analysis of the gut microbiome has identified a protein biomarker that is associated with ageing: a decrease in tryptophan and indole synthesis as a consequence of a decline in the *phylum Firmicutes* in older individuals (> 54 years) (Ruiz-Ruiz et al. 2020).

Ageing patterns within the gut microbiome could have significant clinical implications if beneficial interventions can be identified (Wilmanski et al. 2021). However, whether microbiome diversity and enriched beneficial bacteria are the cause or effect of healthy ageing is still an open question. The utility of including microbiome data in ageing scores also needs to be established. In a recent longitudinal study, microbiome features were informative for mortality risk but did not improve prediction relative to other covariates such as age, sex, BMI, smoking, diabetes, cardiovascular health, and medications (Salosensaari et al. 2021). Also, geography and ethnicity play an important role in microbiome composition (Kong et al. 2016), which may limit the applicability of microbiome biological ageing scores across different countries and cultures.

### Multi-omics biological age

Biological ageing is a complex and multivariate process, and it is unlikely that a single biological data type can quantify every facet of the ageing process. Furthermore, there is a notable lack of agreement amongst different approaches to quantifying BA, suggesting that different biological clocks may be measuring different aspects of ageing (Belsky et al. 2018; Robinson et al. 2020; Vetter et al., 2022b). This has given rise to the hypothesis that clocks compiled from multiple data types may better evaluate BA and more accurately define ageing trajectories than individual data types (Table 4). In a recent longitudinal study, authors performed deep phenotyping of 3558 individuals that included metabolomics, proteomics, genomics, and clinical variables (Earls et al. 2019). The variables most strongly associated with BA were plasma protein levels related to metabolic health, inflammation, and bioaccumulation of toxins. Interestingly, the association of these biomarkers with BA was gender-specific. Notably, this multi-omics approach was sensitive to changes in lifestyle, with a decrease in BA detected amongst participants who were taking part in a wellness program that comprised lifestyle coaching on exercise, nutrition, stress management, and sleep (Earls et al. 2019; Zubair et al. 2019).

Another recent multi-omics study from Ahadi et al. tracked 106 healthy individuals over 4 years. Deep phenotyping - including proteomics, metabolomics, transcriptomics and microbiomics - revealed that each individual had a specific molecular ageing pattern, which the authors termed an “ageotype” [(Ahadi et al. 2020), Table 4]. Ageotypes could be broadly grouped into four categories: liver dysfunction, kidney dysfunction, metabolism and inflammation, and immunity pathways. Inter-individual variability was detected from a relatively young age, which suggests that it may be difficult to create a global ageing score. However, categorization by ageotype could provide a molecular assessment of an individual’s ageing quality, that might prove useful in monitoring and intervening in the ageing process. Longer-term follow-up will be required to determine whether ageotypes can predict changes in organ function over time.

Finally, Zimmer et al. recently combined health questionnaires with longitudinal multiomics data [(Zimmer et al. 2021), Table 4] to create a multi-dimensional health model. Based on clinical data, the authors identified four archetypes/wellness states within the study population. These archetypes were subsequently enriched with omics data to characterise each archetype further. Using an individual’s longitudinal data, the authors found that movement over time within the multidimensional model space could (1) detect transitions of ageing, (2) detect transitions from health to disease, and (3) identify aberrant health conditions.

These multidimensional multi-omics models are complex and unlikely to find practical application in the clinical setting. However, the results of deep phenotyping in exploratory studies will help refine the discovery of new and improved biomarkers of ageing and health in ageing.

## Discussion and future perspectives

Measurements of healthy ageing are a valuable tool for understanding ageing dynamics within and amongst populations. By characterizing populations and their health/longevity outcomes it is possible to expand the knowledge of lifestyle habits or environmental conditions that contribute to the healthspan. Future improvements in ageing quality will require both individual and policy-level changes. To understand which interventions mostly improve ageing quality by increasing homeodynamic space and intrinsic capacity, it is first necessary to understand the variables and underlying mechanisms that contribute to healthy ageing.

Biological ageing is a multidimensional process, and probably no single measurement is capable of quantify all of its aspects. Ageing scores typically measure loss of functionality, i.e. physiological ageing, and are used in different settings but mostly to predict morbidity, disability and mortality. Until recently, methodologies and variables used in ageing studies relied mainly on the functional and societal aspects of ageing with scarce application of molecular measurements (Dato et al. 2021; Stanziano et al. 2010). Using new tools of biological age measurements simultaneously with classical ageing scores, scientists could more quickly determine the utility of these molecular biomarkers (Levine 2020; Oblak et al. 2021).

### Equivalence between ageing measurements

The low equivalence among approaches used for measurement of biological ageing observed in some studies, reveals that each might be quantifying different aspects of the ageing process (Fiorito et al. 2021; McCrory et al. 2020; Vetter et al. 2019; 2022b). An example is the assessment of BA by metabolomics, which was revealed to be complementary, but not associated with established epigenetic clocks, showing an association with distinct lifestyle risk factors instead (Robinson et al. 2020). A comparison of 9 BA methodologies (telomere length, 4 DNA methylation clocks, physiological age, cognitive function, functional ageing index (FAI), and frailty index (FI) was performed in a single longitudinal cohort (Li et al. 2020). All BAs were correlated with each other to some degree, in large part due to their correlations with CA. However, except for telomere length, they were also independently associated with mortality risk, showing that BA can be better than CA at predicting mortality. Of the BA methodologies that were compared, the best independent predictors of mortality were *DNAmGrimAge* and FI. In a joint model, *DNAmHorvath*, *DNAmGrimAge*, and FI showed complementarity in predicting mortality risk.

The differences in outcome prediction when using different methodologies, may arise due to differences in sample size, study-specific age cut-offs to define the affectation status, sex- specificity, and population specificity, i.e., genetic and/or lifestyle heterogeneity among cohorts (Dato et al. 2021). The inclusion of different population backgrounds is particularly crucial in ageing, since it is heavily influenced by a strong geographical component and environmental exposure. The lack of homogeneity in the data obtained from epidemiological, demographic, and even clinical markers is problematic. Also there is limitation to the range of markers obtained from each study (Kwon and Belsky 2021) since they are a combination of multiple assays and sometimes different laboratory methodologies. The ATHLOS project (https://athlos.pssjd.org/) and Maelstrom research catalogue (https://www.maelstrom-research.org/) are examples of resources intended to harmonize data across studies in order to obtain universal scientific data, applicable worldwide. While the universality of ageing scores is not established, researchers must be familiar with the advantages and drawbacks of the different measurements for answering their research questions (Nelson et al. 2020).

Epigenetic clocks are promising measures of ageing quality, that have demonstrated potential to serve as a reliable ageing biomarkers. They are generated from a single multiplex array and include the same measurements across studies making comparisons and validation easier (Kwon and Belsky 2021). However, these DNAm-based biomarkers tools are still not considered a replacement for validated measures of physical and cognitive performance in old age (Maddock et al. 2020). Also, to understand the molecular origins underlying the observable epigenetic differences further investigation is needed.

However, although a lot of progress has been made in identifying biological markers of ageing, the use of molecular biomarkers in ageing scores remains fundamentally challenging. First, the contribution of each molecular biomarker to BA is small, with high variability and frequent replicability issues. Second, molecular biomarkers can be modified in response to multiple factors including genetics, lifetime exposome, and the presence of age-related diseases. Thus, interpreting their significance with respect to ageing can be complex. Third, validating molecular biomarkers as surrogates for health span will require evidence that these scores are modifiable through intervention and that the resulting phenotypes have improved long-term outcomes.

### Use of MLin the measurements of health in ageing

Critics state that the use of ageing scores, especially biological ageing, reduces the comparison of complex biological states, such as the heterogeneity observed in ageing, to the comparison of single numbers, which destroys information because it assumes that age-dependent differences between individuals can be depicted by a single dimension (Freund 2019). Another challenge is that the combination of molecular and phenotypic data is not able to distinguish between the effects and the causes of ageing (Newman, 2015), with sometimes the presence of biomarkers of chronic diseases associated with ageing, being the main drivers of the scores.

An attempt to overcome these limitations is using modern analytic techniques to perform high-dimensional analysis, more representative of biological reality (Cohen et al. 2019). The use of machine learning algorithms for assessing ageing quality allows for the inclusion of more ageing manifestations as outcomes, which may improve the predictive value of the models (Sun et al. 2021). ML allows an hypothesis-free datamining, instead of an hypothesis-driven data testing (Hägg et al. 2019). Given these advantages the application of these models to ongoing ageing cohorts is being implemented more routinely (Gomez-Cabrero et al. 2021; Speiser et al. 2021; Varzaneh et al. 2022).

Recent reviews have approached the challenges associated with integrating omics measurements and ML data analysis in ageing research, calling out to data integration, interpretation and sharing of high-throughput data as the main issues to be resolved (Dato et al. 2021; Zhavoronkov et al. 2019). Despite ML offering an alternative to traditional approaches for modelling outcomes in ageing, scepticism over these methods persists due to lack of reproducibility and interpretability of the complex algorithms that underlie these models (Speiser et al. 2021). Although promising, ML algorithms warrant further characterization and validation, since their biological, clinical and environmental correlates remain largely unexplored (Gialluisi et al. 2022).

### Application of ageing scores in younger populations

**Fig. 1:**
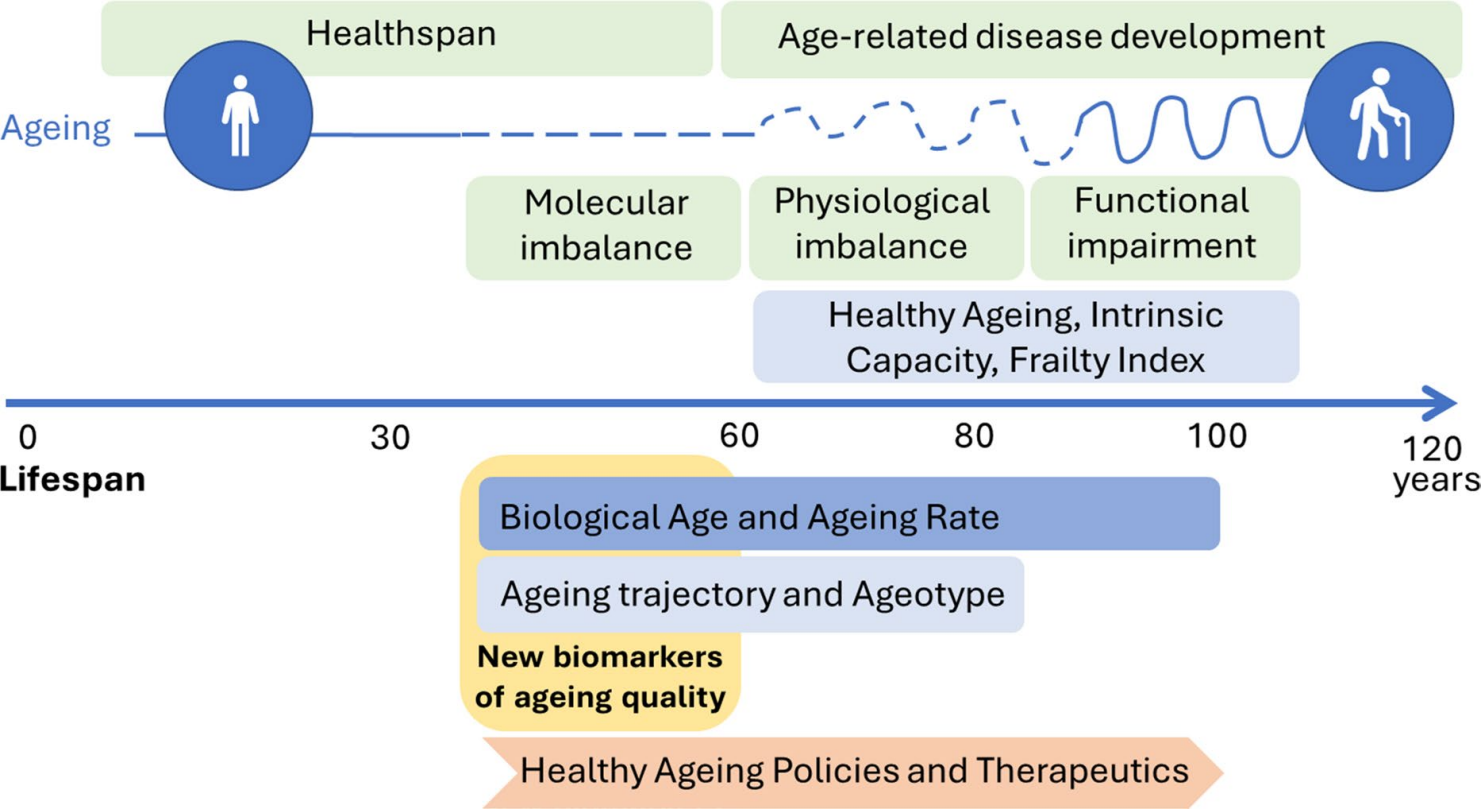
Evaluation of the ageing quality throughout the lifespan. Currently, measurement and evaluation of ageing begin when ageing-related diseases arise, ending the health span period of life. This usually occurs after 60 years of age when physiological imbalance gives rise to functional impairment. Current evaluation of ageing uses several approaches, among them the healthy ageing index, intrinsic capacity construct, and frailty index. In fact, ageing begins earlier in life with the molecular imbalance; application of new biomarkers of ageing quality (ex., epigenetic clocks, transcriptome or metabolome) can be used from early adulthood to determine biological age and ageing rate. In addition, ageing trajectories and ageotype could be used to monitor ageing progression and allow implementation of healthy ageing policies from a young adult age

While phenotypic and physiological ageing scores are excellent tools for assessing ageing quality in the elderly, they have lower utility for predicting ageing quality in younger populations (Nelson et al. 2020). This gap can potentially be addressed by biological ageing scores, thereby enabling the study of early interventions to favour healthy ageing trajectories in a precision medicine scenario (Fig. 1).

The observation that younger adults show variable ageing rates and ageotypes (Ahadi et al. 2020; Belsky et al. 2022; Dieteren et al. 2020; Karimi et al. 2019) makes a strong case for longitudinal studies of biological ageing scores in younger populations. This would allow for the identification of key molecular mechanisms of ageing before the emergence of age-related diseases. The *DunedinPace* clock (Belsky et al. 2022), which was derived from a cohort of young adults followed until the age of 45, showed sensitivity to changes in individual ageing trajectories. However, the ageing outcomes of these individuals, for the next 30 years, still need to be established to understand the relationship between early ageing trajectories and healthspan. Further investigation is needed to understand the cellular and molecular processes that underlie the epigenetic changes of ageing and the redout of the clocks to evaluate ageing quality (Bell et al. 2019; Oblak et al. 2021; Raj and Horvath 2020).

## Conclusion

In the last 5 years, the measurement of healthy ageing has taken a significant leap forward. On the one hand, there has been the development of the concept of intrinsic capacity, recognizing the importance of lifestyle, well-being, and societal participation in achieving healthy ageing. On the other hand, life scientists are plunging ever deeper into molecular measurements of ageing, trying to establish new biomarker panels to identify ageing trajectories and phenotypes. To tackle the current and future challenges of an ageing population, robust ageing scores that encompass all of the alterations suffered by an individual during ageing are required. The ideal HAS should be multisystemic, predictive of future health status, and responsive to change, thereby capturing an individual’s current and future ageing trajectories. It is likely that HAS will differ between the research environment, where in-depth phenotyping is possible and desirable, and the clinical environment, where a more pragmatic approach is required. However, the ideal healthy ageing score for both research and clinical purposes will probably adopt a multi-omics approach to optimize reliability and ensure that the complexity of the ageing process is adequately captured.

## Glossary

**Ageing**: Multifactorial process characterised by functional deterioration, physiological damage, and multiple age-related diseases.
**Ageing phenotype**: Set of measurable ageing traits shared by a population.
**Ageing Scores**: Set of criteria used for the evaluation of ageing quality.
**Ageing trajectories**: The behaviour of ageing phenotypes over time.
**Ageotype**: Ageing patterns that are classified based on molecular pathways that change over time within an individual.
**Biological Age (BA)**: Age measured according to biomarkers/physiological parameters. The difference between chronological age (CA) and biological age is considered a measure of ageing quality.
**Exposome**: Sum of environmental exposures during an individual's lifetime (such as lifestyle behaviours, pollution, or stress).
**Frailty index (FI)**: An age-related condition of increased risk for adverse health outcomes caused by a decrease in homeodynamic space exposing the individual to a higher risk of adverse outcomes, such as multimorbidity, falls, disability, nursing home placement, and death.
**Healthy ageing**: The process of developing and maintaining functional abilities that enable well-being in older age.
**Healthspan**: Period of life spent in good health, free from chronic disease and disability.
**Hormesis**: Mild stress induced-activation of adaptive and protective pathways in cells and organisms, presenting numerous health-promoting, ageing-modulatory and lifespan-extending effects.
**Intrinsic capacity (IC)**: An individual's biological capacity based on five functional domains: locomotion, cognition, psychology, vitality, and sensory. IC goes beyond genetics and health status to encompass how the person functions in their environment.
**Lifespan**: The time between birth and death of an organism.
